# Using Twitter to mine sleep related information from people who declare a diagnosis of a psychotic disorder

**DOI:** 10.23889/ijpds.v1i1.370

**Published:** 2017-04-19

**Authors:** Mladen Dinev, Maksim Belousov, Rohan Morris, Natalie Berry, Goran Nenadic

**Affiliations:** 1 University of Manchester

## Objectives

Our group has investigated the occurrence of psychotic(-like) experiences (PLEs) in Twitter posts, namely auditory hallucinations. Tweets classified as potentially related to auditory hallucinations were proportionately higher between 23:00 and 5:00 in comparison to tweets not classified. This may indicate a clinically significant relationship between sleep and PLEs in the general population, a notion supported by the literature. Based on our previous investigation, the current study aimed to explore whether this methodology could be amended to generate datasets regarding sleep experiences in people who self-report a diagnosis of a psychotic disorder. 

## Approach

The current investigation seeks to establish if it is feasible to generate anonymised datasets regarding sleep by extracting information from the timelines of people who self-report a psychotic diagnosis. A text mining method was implemented that utilised rule-based semantic filters that aimed to identify self-reported diagnoses. This focused on occurrences of personal and possessive pronouns to detect the subjectivity of tweets, as well as potential diagnostic verb indicators and any mentions of other related factors. For each diagnostic tweet, we collected information from user timelines. A sleep-related classifier was then implemented, which used lexical features (e.g. bag-of-words, part-of-speech tags) to predict whether a given tweet refers to sleep-related experience.

## Results

After training the classifier on the bag-of-words model, the most informative words which contributed to the performance of the classifier were: `sleep', `can’t awake', `never', `stress'. Part-of-speech tags (e.g. verbs, adverbs) were also important features. The classification accuracy of the `bag-of-words' model was better than the `part-of-speech' model.

Through the method outlined herein, we were able to improve the quality of the generated datasets in comparison to the previous investigation. This methodology also facilitated the mining of individual Twitter users timelines who stated a personal diagnosis. To this end, an additional filter was implemented to identify tweets regarding sleep experience. The potential relationship between sentiment and temporality expressed in diagnosis and sleep experiences are also discussed. 

## Conclusion

The results from this study have implications for mental health research on Twitter. Specifically, the refinements in the methodology enabled retrieval of two high quality datasets regarding psychosis and sleep. Therefore it is feasible other psychosis-related phenomena (e.g. visual hallucinations, delusions, medication) could also be applied as separate filters to create one dataset of psychosis-related experiences within individuals diagnosed with psychosis.

